# Encephalopathy and COVID-19: a case report

**DOI:** 10.11604/pamj.2021.38.139.27845

**Published:** 2021-02-08

**Authors:** Mohamed Lazraq, Sabah Benhamza, Soufiane Saadaoui, Salah Hayar, Mounir Louardi, Hind Moujahid, Abdelhak Bensaid, Youssef Miloudi, Najib El Harrar

**Affiliations:** 1 Intensive Care Unit, 20 August Hospital 1953, University Hospital Center IBN Rochd, Casablanca, Morocco,; 2 Hassan II University, Faculty of Medicine and Pharmacy, Casablanca, Morocco

**Keywords:** Encephalopathy, SARS-CoV-2, without respiratory distress, case report

## Abstract

Case numbers reported in literature with neurological manifestations potentially linked to COVID-19 is constantly increasing. Most often it is sudden anosmia, headache, encephalopathy or stroke. Pathophysiology of this neurological involvement is still poorly understood. While viral genome is very rarely detected in cerebrospinal fluid, inflammatory involvement is often very significant. We report a case of SARS-CoV-2 encephalopathy without respiratory distress or cytokine storm.

## Introduction

The COVID-19 pandemic has raised several questions about pathogenicity of a virus that continues to surprise medical world with various manifestations. SARS-CoV-2 has been classified as a betacoronavirus like MERS-CoV and SARS-CoV; it infects human cells via the receptor of Angiotensin-converting enzyme 2 (ACE2). Clinical manifestations of COVID-19 are diverse and affect several devices where these receptors are present, and not only respiratory system, this is how neurological, renal, cutaneous, systemic and cardiovascular manifestations have been associated with it. Although the most frequent clinical presentation is obviously respiratory involvement, number of reported cases with neurological manifestations potentially linked to COVID-19 is constantly increasing. We were interested in this impairment through the analysis of our clinical case.

## Patient and observation

**Figure 1:**
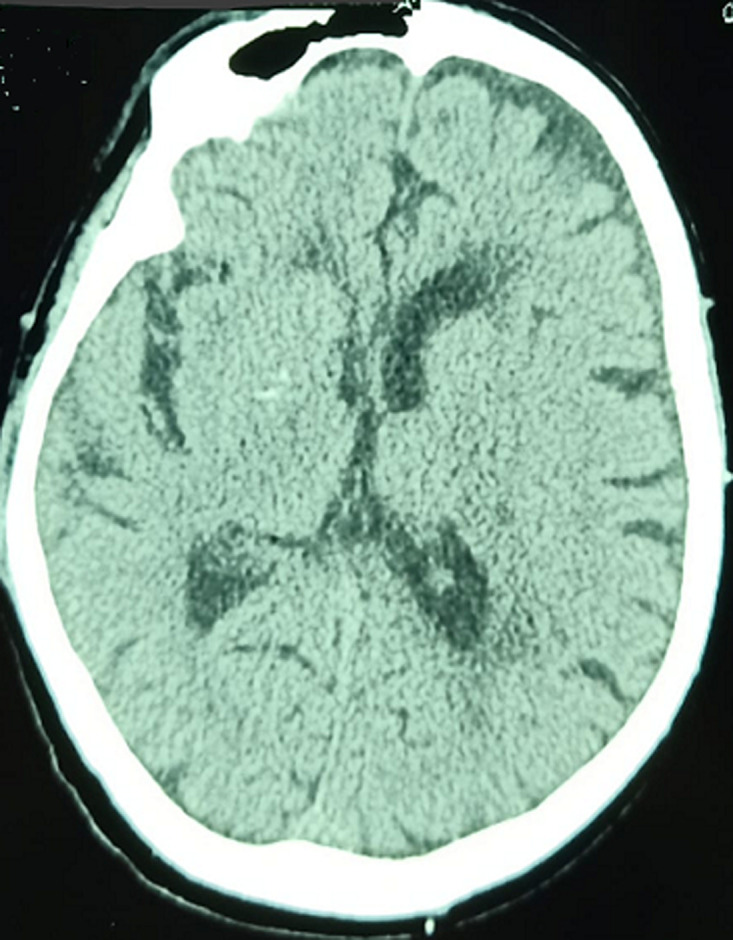
CT scan showing parietal hypodensities

We report the case of a male patient, 79 years old, diabetic for 10 years on metformin, hypertensive for 5 years on amlodipine; occasional alcoholic weaned 4 years ago. He was initially admitted for mental confusion with sudden onset dysarthria dating back 48 hours before. Initial assessment found Glasgow score (GCS) of 12/15 (eye opening at 5, verbal response at 2, motor response at 5), symmetrical and reactive pupils, without feeling-motor deficit, normal osteotendinous reflexes, slightly polypneic at 24 cycles/min, pulsed oxygen saturation (SpO_2_) at 97% in ambient air, he was tachycardic at 115 bpm and hypertensive at 160/95 mmHg, his capillary blood glucose at 2.24 g/L, and glycosuria on the urine dipstick test without ketonuria. The patient was apyretic. Arterial gas testing showed normal corrected anion gap metabolic acidosis, with hypokalaemia at 3 mmol/L and normal lactatemia at 0.43 mmol/L. Diagnosis of diabetes decompensation was made despite absence of ketone bodies on urine dipstick test. After conditioning, the patient was put on a hydration regimen with hypokalaemia correction and insulin therapy, and then referred to intensive care for additional management in face of non-improvement in his neurological condition. A brain scan was performed objectifying multiple parietal ischemic foci (Figure 1), chest scanner showed a thickening of the septal and foci of bronchial dilation without sign of pneumopathy linked to COVID-19 infection; abdominal ultrasound did not show any abnormalities.

**Figure 2:**
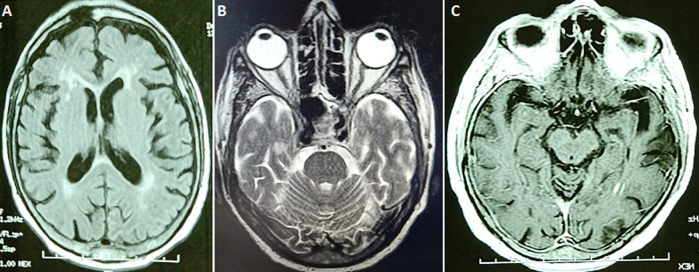
MRI images weighted in T1 (A), T2 (B) and flair (C) showing vascular leukopathy, with some calcifications in basal ganglia

An electrocardiogram was performed showing no electrical signs of hypokalaemia or repolarization disturbances, transthoracic echocardiography was normal, as was ultrasound of supraortic trunks. Laboratory workup showed an elevated C-reactive protein (CRP) of 45.5 with negative procalcitonin (0.04 ng/ml), white blood cells at 12,500 (polymorphonuclear neutrophil (PNN) at 9280 and normal lymphocytes at 2140), no thrombocytopenia, normal ferritinemia, fibrinogen levels elevated to 5.37 and negative D-dimers. Natremia, magnesemia, corrected serum calcium, phosphoremia and thyroid assessment was normal. SARS-CoV-2 reverse transcription polymerase chain reaction (RT-PCR) by nasopharyngeal swab was negative, COVID-19 serology (IgM and IgG) was also negative. Faced with concept of ischemic lesions on a brain scan, anticoagulation at a prophylactic dose, antiplatelet aggregation with aspirin and statins were initiated as well as an antibiotic therapy at a meningeal dose with ceftriaxone pending results of the lumbar puncture which subsequently showed a clear liquid with a high proteinorachia at 0.58g/l (normal between 0.15 and 0.45), a normal glycorachia/blood sugar ratio at 0.58 (normal at 0.5); with a culture showing less than 3 elements, sterile after 24 hours. Cerebral Magnetic resonance angiography performed on day 2 of admission (Figure 2 A, B, C) showed signs in favor of vascular leukopathy, with some calcifications in basal ganglia and cortical atrophy. Electroencephalogram showed signs of epilepsy, which prompted us to retain the diagnosis of nonketotic hyperglycemia-related epileptic seizures. Anticonvulsant treatment was started with sodium valproate. The Patient worsened neurologically despite correction of his metabolic acidosis, he was intubated on day 3 of his admission (day 5 of symptoms onset). A cerebral computerized tomography (CT) scan was performed without showing any progressive lesions. Faced with installation of lymphopenia (HIV serologies 1 and 2 negative) with increased CRP, a multiplex RT-PCR in protected distal bronchial sample as well as in Cerebrospinal fluid (CSF) were performed, the first was positive to SARS-CoV-2. Reverse transcription polymerase chain reaction of SARS-CoV-2 in CSF was positive but with a cycle threshold (CT) of 38.6. The diagnosis of SARS-CoV-2 encephalopathy was retained. The patient is still in intensive care.

## Discussion

Our clinical report is a case of SARS-CoV-2 encephalopathy without severe pulmonary involvement or significant inflammatory syndrome. Severe Acute Respiratory Syndrome Corona Virus-2 (SARS-CoV-2) is an RNA virus that has phylogenetic similarities to SARS-CoV, and both use the same receptors to enter cells, to namely those of angiotensin-converting enzyme 2 (ACE2), these receptors are expressed mainly in lung, enterocytes, venous and arterial vascular endothelium and in arterial smooth muscle cells [1]. They have also been found on glial cells and neurons [2]. Neurological damage observed in COVID-19 can be explained, as is case for several respiratory viruses, by two phenomena, the first being hematogenous dissemination by lesions of blood-brain barrier or by direct invasion via the ethmoid riddled blade close to olfactory bulb, the second is part of cytokine storm [2]. An MRI study carried out post-mortem in 19 previously neurologically healthy patients showed abnormalities suggesting intracranial vascular involvement in 21% of cases as well as asymmetric olfactory bulbs in 21% of cases. These lesions were of the type of micro and macro sub-cortical bleeding predominantly posterior which could be compatible with lesions of disseminated intravascular coagulation promoted by extra-corporeal oxygenation membrane. Another patient had posterior subcortical bleeding; another brain lesion suggestive of posterior reversible encephalopathy syndrome (PRES). The fourth death had hypersignals of semi-oval center at T2 weighted image. All these lesions have in common an attack of vascular endothelium, this corroborates the theory of direct viral infection by ACE2 receptors and that of subsequent induced cytokine storm, both of which may be responsible for such endothelial damage. In 4 patients, an MRI correlation was found between anosmia and an obliteration of the olfactory cleft, despite respiratory distress no brainstem lesion was found on magnetic resonance imaging (MRI), excluding central origin of the distress [3].

In favor of cytokine storm theory, a multicenter study of three hospitals in Wuhan involving 214 COVID-19 patients reported neurological manifestations in 36.4% of cases. This observational study identified three types of neurological involvement, central nervous system (headache, consciousness disorders, strokes, convulsions and ataxia), peripheral nervous system and cranial pairs (dysgeusia, anosmia, peripheral neuropathy) and skeletal muscle damage. These attacks were significantly higher in the group of severe patients with higher D-dimer levels and greater lymphopenia [4]. In our case, the patient did not present with significant inflammatory syndrome making the theory of neurological damage secondary to cytokine storm unlikely in his case. In a French observational study, involving 58 patients with neurological manifestations in COVID-19 caused by SARS-CoV-2, all patients had positive nasopharyngeal RT-PCRs, 7 patients having benefited from a cerebrospinal fluid (CSF) study had no cellularity and RT-PCR was negative in CSF thus eliminating the theory of SARS-CoV-2 diffusion in CSF of these patients [5]. In our case, SARS-CoV-2 was detected in CSF but with a CT (cycle threshold) of 38.6, which raises the question of a possible revision of the threshold of positivity of the PCR in this site and the probability of neurological lesions even with traces of virus in CSF, further studies are needed in order to establish this theory.

## Conclusion

As the COVID-19 pandemic progresses, reports of neurological manifestations are increasing. These manifestations can be considered as direct effects of corovirus on nervous system or as an immune-mediated disease in the setting of cytokine storm. Our case presents an atypia due to negative inflammatory markers and anti-SARS-CoV-2 antibodies as well as a negative PCR in CSF at usual thresholds which raises the need for a systematic search for an infection by SARS-CoV-2 in CSF in front of any atypical neurological picture.
